# An unusual presentation of a malignant jejunal tumor and a different management strategy

**DOI:** 10.1186/1477-7819-3-3

**Published:** 2005-01-09

**Authors:** Atul Samaiya, SV Suryanarayana Deo, Sanjay Thulkar, Sidhartha Hazarika, Sunil Kumar, Dillip K Parida, Nootan K Shukla

**Affiliations:** 1 Department of Surgical Oncology, Institute Rotary Cancer Hospital (IRCH), All India Institute of Medical Sciences (AIIMS), New Delhi 110029, India; 2 Department of Radio-diagnosis, Institute Rotary Cancer Hospital (IRCH), All India Institute of Medical Sciences (AIIMS), New Delhi 110029, India; 3 Department of Radiotherapy, Institute Rotary Cancer Hospital (IRCH), All India Institute of Medical Sciences (AIIMS), New Delhi 110029, India

## Abstract

**Background:**

Malignant small bowel tumors are very rare and leiomyosarcoma accounts for less than 15% of the cases. Management of these tumors is challenging in view of nonspecific symptoms, unusual presentation and high incidence of metastasis. In this case report, an unusual presentation of jejunal sarcoma and management of liver metastasis with radiofrequency ablation (RFA) is discussed.

**Case presentation:**

A 45-year-old male presented with anemia and features of small bowel obstruction. Operative findings revealed a mass lesion in jejunum with intussusception of proximal loop. Resection of bowel mass was performed. Histopathological findings were suggestive of leiomyosarcoma. After 3-years of follow-up, the patient developed recurrence in infracolic omentum and a liver metastasis. The omental mass was resected and liver lesion was managed with radiofrequency ablation.

**Conclusion:**

Jejunal leiomyosarcoma is a rare variety of malignant small bowel tumor and a clinical presentation with intussusception is unusual. We suggest that an aggressive management approach using a combination of surgery and a newer technique like RFA can be attempted in patients with limited metastatic spread to liver to prolong the long-term survival in a subset of patients.

## Background

Malignant tumors of the small bowel are rare and accounts for the <2 % of total gastrointestinal (GI) malignancy [1,2]. The age-adjusted incidence of small bowel malignancy is 1 per 100,000 with prevalence of 0.6% [3]. Management of these tumors is challenging because of their rarity, relative inaccessibility for diagnosis and diverse histologic types and nonspecific symptoms [4]. Because of the heterogenous and aggressive nature many of them present with recurrence and visceral metastasis. Radiofrequency ablation has been successfully tried as a minimally invasive but effective local treatment of liver metastasis from a variety of primary sites including small bowel tumors [5]. In this case report, we present an unusual case of small bowel sarcoma, and discuss the clinical presentation and management using combination of surgery and radiofrequency ablation (RFA).

## Case presentation

A 45-year-old male was referred to our center with the diagnosis of suspected non-Hodgkin's lymphoma (NHL) of the bowel in December 1999. He had generalized weakness for 2-years along with recurrent vomiting, occasional constipation and melaena for last 2 months. The diagnosis was considered after an ultrasound (USG) guided fine needle aspiration cytology (FNAC) from the intra-abdominal mass done elsewhere and showed features suspicious of NHL.

At presentation, patient's general condition was poor and he was dehydrated and pale. There was no peripheral lymphaedenopathy. Abdominal examination revealed an ill-defined, mobile, nontendor lump in left paraumblical region extending up to left lumber region. There was no hepato-splenomegaly. Examination of chest and cardiovascular system was normal. After initial resuscitation with crystalloids and blood transfusion, patient was further investigated.

At presentation his hemoglobin was low (6.4 gm%) but rest of the routine haematological investigations were within normal limit. The chest X-ray was normal. Abdominal ultrasound (USG) showed a left upper abdominal mass lesion suggestive of bowel mass. The upper GI endoscopy was normal. The barium meal follow through examination was suggestive of intussusception of proximal jejunum and a suspected mass lesion at the leading edge of intussusceptum. An USG guided core needle biopsy of mass was performed because of earlier suspicion of NHL, which showed smooth muscle bundles with areas of necrosis. Based on the above findings and biopsy report, patient was taken up for exploratory laparotomy 3 weeks after initial presentation.

Operative findings revealed an intussuscepted proximal jejunum loop 12 cm distal to duodeno-jejunal flexure and a vascular polypoidal growth measuring 6.5 × 5 × 3.5 cms on serosal surface of jejunum. Liver and spleen were normal. There was no evidence of mesenteric or retroperitoneal lymphaedenopathy, ascites or peritoneal disease. The resection of involved segment of jejunum with 5 cm margins and an end-to-end anastomosis was performed. The postoperative course was uneventful.

**Figure 1:**
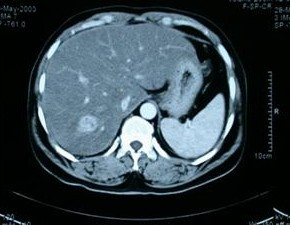
CT scan image showing the hepatic metastasis in segment VII.

**Figure 2:**
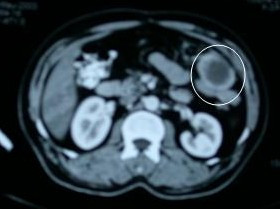
CT scan image showing the omental mass adjacent to bowel loop (circle around the omental mass).

Pathological examination of the specimen revealed two mass lesions measuring 4.5 cm & 3 cm in continuity (total 7.5 cm in maximum diameter) located on mucosal and serosal surface of jejunum respectively. Microscopically it showed spindle cell tumor with a mitotic rate of 5/10 high power fields (HPF). The tumor was negative for desmin, S-100, CD 34 and c-kit (CD117) but focally positive for actin on immunohistochemistry (IHC). The resection margins were free. The final diagnosis of malignant spindle cell tumor – leiomyosarcoma of jejunum was made. Due to negative margins and no evidence of disease elsewhere, no adjuvant therapy was planned and patient was kept on regular follow up. He was assessed clinically at three months interval and an USG of abdomen was performed six monthly. Patient remained disease free for 39 months. After that a follow-up USG showed a space-occupying lesion (SOL) in segment VII of liver and a mass lesion in left upper abdomen. The patient was asymptomatic and abdominal examination revealed no abnormality. A computerized tomographic (CT) scan of abdomen was performed which showed a 2 × 2 cms, brightly enhancing SOL in segment VII of liver (Figure 1) and a 3 × 4 cms omental mass lesion abutting the bowel loop in left upper abdomen (Figure 2). Upper GI endoscopy and colonoscopy did not reveal any abnormality. A provisional diagnosis of recurrent leiomyosarcoma was made. Patient was planned for resection of the mass and RFA of liver lesion.

Patient was explored and two separate well encapsulated, fleshy mass lesions were found in infracolic omentum, measuring 6 × 7 × 3.5 and 2 × 1 × 1 cms. The liver lesion was not palpable and there was no evidence of disease at primary site or anywhere else in peritoneal cavity. An infracolic omentectomy along with tumor nodules was performed.

**Figure 3:**
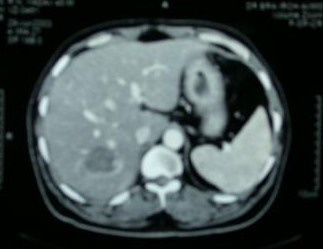
Post radiofrequency ablation CT scan image showing complete resolution of hepatic metastasis.

The histopathological features were suggestive of a highly cellular tumor composed of spindle shaped cells arranged in interlacing fascicles. On immunohistochemistry, multiple sections were negative for CD-34, CD-117, S-100 and desmin but focally positive for actin as in the initial tumor. So a diagnosis of recurrent leiomyosarcoma was made. The patient was taken up for an USG guided RFA of liver metastasis 3 weeks after surgery. The RFA was performed by Cool Tip™ RF machine [Radionics, USA] using single tip probe. The procedure was conducted for 12 minutes at temperature of 65°C and the lesion was ablated with 1 cm margin. Follow-up CT scan done 3 days later showed complete ablation of metastatic lesion (Figure 3). The patient was kept on regular follow-up and nine months after RFA he was asymptomatic and an abdominal CT scan showed no evidence of recurrence.

## Discussion

The small intestine accounts for 75% of total length of GI tract and more than 90% of mucosal surface. However, it is the site of only 6–25% of GI neoplasms and only 2% of GI malignancies [2,6]. Leiomyosarcoma is the fourth most common malignant tumor of small bowel and it's incidence is 1.2 cases/million/year [7,8].The most frequent site of leiomyosarcoma is jejunum, followed by ileum and duodenum [6,8]. The peak incidence is in 6^th ^decade and there is a slight male preponderance [6].

Most of these tumors are slow growing and associated with a long period of symptoms [8]. The most common presentation is GI bleeding and anemia [6]. Abdominal pain and palpable mass is present in 5–50% of patients [6]. Other symptoms include nausea, vomiting and weakness [7]. Nonetheless, there is no specific sign or symptom that defines presentation of smooth muscle tumors [7]. Our patient presented with features of GI bleed, anemia and recurrent subacute intestinal obstruction.

Intestinal obstruction is seen in less than 5% patients with smooth muscle tumor and is usually caused by tumor infiltration or malignant adhesions [6,7]. In the present case, the cause of obstruction was intussusception. In adult patients, small bowel intussusception is caused mostly by small benign intraluminal ileal tumors like lipoma and leiomyoma [6,8]. However, in this patient intraluminal component of dumb bell jejunal leiomyosarcoma was causing intussusception. Intussusception is a rare phenomenon in patients with leiomyosarcoma and tumors of ileal region presents with intussusception more often than jejunal location [6]. Review of English literature revealed only one case of jejunal intussusception caused by leiomyosarcoma [9]. The commonly recommended investigations in cases of suspected small bowel tumor are endoscopy, CT scan and contrast studies and most often the final diagnosis is confirmed after laparotomy and histopathology [6] as happened in the present case. The preoperative tissue diagnosis is not routinely recommended except in suspected cases of lymphoma, germ cell tumor and unresectable metastatic disease because of theoretical risk of peritoneal seeding and tumor rupture along with difficulty in obtaining definitive diagnosis [10,11].

Majority of leiomyosarcoma grow extraluminally but infrequently tumor can grow both extra and intraluminally. Blanchard *et al *in their extensive review of small bowel leiomyosarcoma found that 68% of jejunal tumors grow extraluminally and only 14.4% in dumb bell fashion [6]. Tumor size varied from 1–20 cm and in >3/4^th ^of patients tumor is >5 cm. In the present case, the tumor was 7.5 cms and had grown in a dumb bell fashion.

The surgical resection is the mainstay of treatment in these tumors [6,7]. The surgery usually involves the *en bloc *resection of tumor with wide margins along with adjacent mesentery [6]. The lymphatic spread of leiomyosarcoma to regional lymph nodes occurs in 5%–13% of patients and role of lymph node dissection is controversial. However, routine lymph nodal dissection in the absence of nodal disease is not recommended [7].

Unfortunately, leiomyosarcomas are notoriously radioresistant and attempts at using adjunctive radiotherapy and chemotherapy have failed [12,13]. There is no role of adjuvant therapy and patients with complete resection are kept on close follow-up [6,7,13]. Periodic USG or CT scan is recommended, as biologically these tumors are heterogeneous and aggressive in nature [10]. Similar guidelines were followed in this patient.

The differentiation between benign and malignant smooth muscle tumors is difficult and metastasis is the only conclusive evidence of malignancy. Similar to other sarcomas, the most common route of metastasis is hematogenous and common sites are liver and lungs. But unlike other sarcomas peritoneal seeding is more commonly seen [6]. Blanchard *et al *reported 41.3% incidence of metastasis in jejunal leiomyosarcoma and liver was the most common site followed by peritoneum and mesentery [6]. They also found recurrent tumors in 40.9% of patients in conjunction with metastatic disease. A similar picture was present in our patient.

Ranchod and Kempson [14] reported that number of mitosis is the single most criterion for predicting the metastatic potential and tumors with ≥5 mitosis per 10 high power fields (HPFs) behaved more aggressively. A tumor that shows >2 mitosis/HPF is usually considered malignant [6]. The size of tumor is also considered in risk stratification for metastasis and tumors <5 cms are significantly less likely to metastasize [6]. With this criterion, our patient falls into high-risk group for relapse.

Overall reported survival rate for GI leiomyosarcoma is 10–48% [6]. But in an analysis of six series, Licht *et al *[15] reported only 5–20%, 5-year survival for high-grade tumors (tumors with >10 mitosis per 10 HPFs). O'Riordan *et al *[8] reported 5-year survival rate of 27% for tumors >5 cms. In a series of 21 patients with high grade leiomyosarcomas, Chou *et al *found that 17 of their cases had liver metastasis or local recurrence [16].

Conlon *et al *[17] reported 44% recurrence after complete resection, and mean time to recurrence was 9 months (median 7, ranging from <1 to 37 months). Despite the high-risk, our patient had relapse after 40 months of primary surgery and the relapse sites were omentum and liver, without having any evidence of disease at primary site.

A few patients benefit from surgical intervention for removal of metastasis in small bowel leiomyosarcoma [7]. Karakousis *et al*, [18] reported prolongation of survival after metastasectomy in intestinal leiomyosarcoma and 3 year survival of 28%. Kohno *et al *[19] described 10-years survival after multiple surgeries in a patient of intestinal leiomyosarcoma. Considering the good general condition of our patient, long disease free interval and limited bulk of disease, we planned for resection of omental recurrence and treat the liver lesion with RFA.

Most of the experience of liver metastasis management is in colorectal malignancy and there is limited experience of liver resection in noncolorectal cancers [20]. There are different types of therapy for the management of liver metastasis – surgical resection, regional treatment modalities, systemic therapy and local or *in situ *ablation [21]. Although, the gold standard is surgical resection however only 15–25% of patients with liver metastasis are suitable for hepatic resections [20,21]. In soft tissue sarcoma; in a selected group of patients, metastatectomy is recommended [22].

Due to high morbidity and mortality and limited survival after liver resection, recently, *in situ *ablation of liver metastasis with radiofrequency ablation is gaining popularity [5,20,21]. RFA is ideally suitable for single lesion <5 cm in size and complication rate of RFA is usually very low [23]. The effectiveness of RFA has been demonstrated in few retrospective studies but long-term results are awaited [24].

Considering the very high-risk of relapse in leiomyosarcoma and a deep-seated liver metastasis requiring major hepatic resection, we planned for the RFA of liver lesion. Until now, there are only a few reports of use of RFA in treatment of liver metastasis in leiomyosarcoma [25,26]. Tepetes *et al *[25] reported 5-year survival in a case of hepatic metastasis from leiomyosarcoma with combination of liver resection, RFA and systemic chemotherapy.

Karakousis *et al *[18] reported 3 and 5 years survival as 28% and 4% respectively after complete resection of metastasis in intestinal leiomyosarcoma. However, survival was limited to 6 months for high-grade leiomyosarcoma. Despite a high-risk tumor and having local relapse with liver metastasis, our patient is still disease free after 9 months of second surgery and 4 years after first surgery.

## Conclusion

Jejunal leiomyosarcoma is a rare variety of malignant small bowel tumor with diverse presentation, heterogeneous behavior and a high propensity for relapse. Clinical presentation with intussusception is unusual. We suggest that an aggressive management approach using a combination of surgery and a new technique like RFA can be attempted in patients with limited metastatic spread to liver to prolong the long term survival in a subset of patients.

## Competing interest

The author(s) declare that they have no competing interests.

## Authors' contributions

**SVSD, NKS: **Surgical management and review of manuscript.

**AS, SH, SK: **Review of literature and preparation of manuscript.

**ST, DKP: **Radiofrequency ablation.

All authors have read and approved the contents of manuscript.
